# Maternal perception of child weight and concern about child overweight mediates the relationship between child weight and feeding practices

**DOI:** 10.1017/S1368980022000040

**Published:** 2022-07

**Authors:** Jian Wang, Daqiao Zhu, Xuwen Cheng, Yicong Liuzhou, Bingqian Zhu, Scott Montgomery, Yang Cao

**Affiliations:** 1 Shanghai Jiao Tong University School of Nursing, 200025 Shanghai, People’s Republic of China; 2 Florence Nightingale Faculty of Nursing, Midwifery & Palliative Care, King’s College London, London, UK; 3 Department of Children’s Disease Prevention, Jinyang Community Health Service Center, Shanghai, People’s Republic of China; 4 Department of Nursing, Shanghai Children’s Medical Center, Shanghai, People’s Republic of China; 5 Clinical Epidemiology and Biostatistics, School of Medical Sciences, Örebro University, Örebro, Sweden; 6 Clinical Epidemiology Division, Department of Medicine, Karolinska Institutet, Stockholm, Sweden; 7 Department of Epidemiology and Public Health, University College London, London, UK

**Keywords:** Preschool children, Maternal concern, Weight perception, Feeding practices

## Abstract

**Objective:**

To examine the mediating effects of maternal perception of child weight (weight perception) and concern about overweight (weight concern) on the paths between child weight and maternal feeding practices.

**Setting:**

Pudong District, Shanghai, China.

**Participants:**

A convenience sample of 1164 mothers who were primary caregivers of preschool children.

**Results:**

Sixty per cent of the mothers perceived their overweight/obese children as normal weight or even underweight. The disagreement between actual child weight and maternal weight perception was statistically significant (Kappa = 0·212, *P* < 0·001). Structural equation modelling indicated that weight perception fully mediated the relationship between child BMI Z-scores and pressure to eat. Weight concern fully mediated the relationships between child BMI Z-scores and the other three feeding practices. The serial mediating effects of weight perception and concern were statistically significant for the paths between child BMI Z-score and monitoring (*β* = 0·035, *P* < 0·001), restriction (*β* = 0·022, *P* < 0·001), and food as a reward (*β* = –0·017, *P* < 0·05).

**Conclusion:**

Child weight may influence maternal feeding practices through weight perception and concern. Thus, interventions are needed to increase the accuracy of weight perception, which may influence several maternal feeding practices and thereby contribute to child health.

Childhood obesity is a worldwide health issue that disproportionally affects certain ethnic groups^(1)^, and its prevalence in Asian is rising^(2)^. In China, childhood overweight prevalence increased from 11·7 % to 25·2 % and obesity prevalence increased from 2·8 % to 10·1 % from 1991 to 2011^(3)^. The overall rate of overweight and obesity among children in China is lower than that of Western countries, but the rate of increase over the last 30 years has not been slower in China^(4)^.

Parental feeding practices may influence the eating habits of young children, and thus appropriate feeding strategies are critical to prevent and control childhood obesity^(5,6)^. Feeding practices refer to specific practices or strategies that parents employ to manage what, when and how much their children eat and shape their children’s eating patterns^(7–9)^. Several feeding practices, such as applying pressure to eat, restricting food, monitoring consumption and using food as a reward^(9)^, have been most frequently studied^(10–13)^.

Many studies have identified factors related to feeding practices^(14–18)^. Among these factors, child weight has been an important factor^(14,16–18)^. Longitudinal studies indicated that mothers of children with higher BMI Z-scores were more likely to apply restrictive feeding practices^(19–21)^ and monitor their children’s diet^(19)^ and less likely to apply pressure to eat^(19,21)^ than mothers of children with lower BMI Z-scores. However, it has been reported that parental perception of and concern about child weight (weight perception and weight concern) rather than actual child weight are associated with parental feeding practices^(22–25)^. For instance, Payne *et al.* ^(24)^ found that parents who were highly concerned about their child’s weight were more likely to use restrictive feeding than parents with lower levels of concern, while there was no significant association between child weight and feeding practices. Yilmaz *et al.* ^(25)^ found that parents were less likely to encourage their children to eat if they perceived them as overweight than if they perceived them as normal weight or underweight. In comparison, Freitas *et al.* ^(26)^ recently reported that maternal restrictive feeding practices were independently associated with mothers’ weight perception and concern as well as their child’s weight status. Overall, current evidence of the relationships between maternal weight perception and concern, actual child weight, and maternal feeding practices has been inconsistent. The inconsistent findings might be due to differences in factors included in the analyses (especially weight-related variables). Some studies did not examine maternal weight perception and/or weight concern and accordingly discovered only correlations between maternal feeding practices and child weight^(16,27)^. Other studies performed univariate analyses to investigate associations between factors of interest and parental feeding practices^(16,25,28,29)^.

The above evidence is mainly from developed countries. The relationships between feeding practices, maternal weight perception and child weight may be very different between developed and developing countries given the differences in feeding cultures and beliefs. In developing countries (e.g. China) or low social economic settings^(30)^, parents do not often perceive overweight or obesity as a health issue^(31,32)^. Notably, most families only have one child due to the One-Child Policy in China^(33)^. Chinese caregivers (parents/grandparents) tend to overfeed their children or feed them with energy-dense food as they believe that higher weight indicates better health^(34,35)^. They have the tendency to use food as a symbol for their love for their children, or as an educational and emotional tool for shaping their children’s behaviours^(36)^. Since parental feeding practices carry cultural variations, there is a need to examine the correlations in the Chinese background, which may add unique contributions to the current knowledge base.

Evidence-based theories of information processing and behaviourism learning, such as Gagne’s Information Processing Model^(37)^, have suggested that cognitive changes (e.g. perception or concern) occur only when an objective stimulus (e.g. child weight) exists. The changes may then lead to specific behaviours (e.g. feeding practices). Similarly, Mareno proposed a middle-range explanatory theory in which the following elements occurred in order: parental beliefs and values about child body weight, parental perception of child weight, parental concern and family lifestyle changes^(38)^. Therefore, caregivers’ weight perception and weight concern might mediate the association between child weight and feeding practices. A study of 7- to 9-year-old children in the UK reported that maternal concern about overweight fully mediated the relationship between child adiposity and the use of restrictive feeding practices^(39)^. In addition, previous research has indicated that child weight plays an important role in caregivers’ feeding practices^(14,16–18,26)^.

Based on the above theories and empirical evidence, we proposed a hypothesised model (Fig. 1). In this model, (1) child weight had a direct effect on four common feeding practices, and (2) the associations between child weight status and these feeding practices were mediated by maternal weight perception and weight concern. To explore the relationships proposed in the hypothesised model, we used structural equation modelling while controlling for potential covariates, such as child age^(15,17)^ and sex^(15,17)^. We also controlled for caregivers’ age^(17,40)^ and weight classification based on BMI^(17,18)^, education level^(15,18)^ and family income^(40,41)^.

**Fig. 1:**
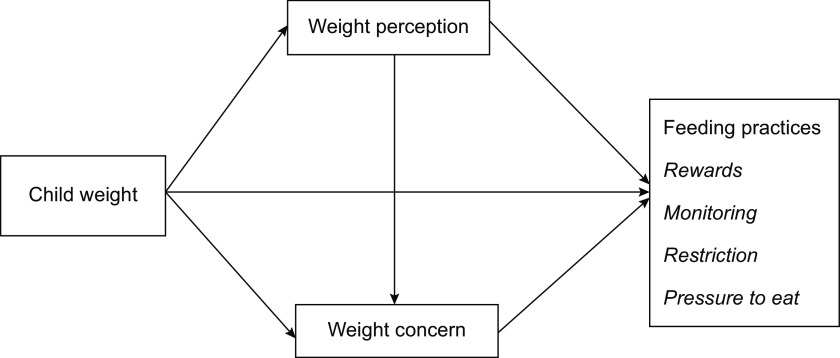
Hypothesised model for relationships between variables of interest. Weight perception: maternal perception of child weight; weight concern: maternal concern about child overweight

## Materials and methods

### Study design and participants

A correlational and cross-sectional design was used. This study was conducted between December 2017 and May 2020 in Shanghai, China. A convenience sample was selected from three public kindergartens located in three areas representing different economic levels in Pudong District, Shanghai. Only mothers were recruited because they are the primary caregivers of children typically in China. The required minimum sample size was 346, calculated using the formula below^(42)^:

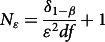

where *α* = 0·05, *β* = 0·90, *ϵ* = 0·05, *δ*_1–*β*_ ≈ 33·6 and *df* = 39.

A total of 1164 mothers were recruited. We excluded unqualified questionnaires, mothers who were not the primary caregivers and children with diseases related to nutrition or extreme age-standardised BMI Z-scores. Finally, 1106 (95·02 %) responses were included in this report.

### Demographic and socio-economic data

Demographic and socio-economic data, including children’s age, sex and mothers’ age, weight, height, education level, and annual household income, were measured using a self-reported questionnaire. Children’s weight and height were measured and collected by trained health teachers in the kindergartens. Maternal weight status was classified as underweight (BMI < 18·5 kg/m^2^), normal weight (18·5kg/m^2^ ≤ BMI < 24·0 kg/m^2^), or overweight or obese (BMI ≥ 24·0 kg/m^2^)^(43)^. According to the WHO guidelines, child age-standardised BMI Z-scores were calculated using the software WHO Anthro (for 2- to 5-year-old children) and Young Growth Curve (for 5- to 6-year-old children). BMI Z-scores were categorised into three groups: underweight (Z-score < –2), normal weight (–2 ≤ Z-score ≤ 1), and overweight or obese (Z-score > 1)^(44)^.

### Maternal feeding practices

Maternal feeding practices were evaluated using the Chinese version of the Child Feeding Questionnaire (C-CFQ)^(45)^. The culture-specific feeding items to assess parental using food as a reward were added to the C-CFQ, which has showed better factor structures than the initial instrument developed by Birth *et al.* ^(46)^ among Chinese samples. The C-CFQ assesses four types of feeding practices: four items of monitoring (the extent to oversee their child’s eating^(46)^), six items of restriction (strict limitations on the child’s access to foods or opportunities to consume unhealthy foods^(47)^), four items of pressure to eat (insists, demands or physically struggles with the child in order to get the child to eat more food^(46–48)^) and two items of food as a reward (use of desired foods as a way to regulate the child’s eating or behaviours^(48)^). Each item was rated on a five-point Likert scale. The response options for each item were ‘always,’ ‘usually,’ ‘sometimes’, ‘almost never’ and ‘never’. Each subscale was calculated by averaging the scores of all the items in that subscale. The C-CFQ demonstrated good internal consistency reliability in the current study (Cronbach’s α = 0·748–0·890). The questionnaire also showed good construct validity^(49,50)^.

### Maternal perception of child weight (weight perception) and concern about overweight (weight concern)

Weight perception was assessed by asking ‘How would you describe your child’s weight?’. The responses included ‘very underweight’, ‘slightly underweight’, ‘normal weight’, ‘slightly overweight’ and ‘very overweight.’ Weight concern was assessed by asking ‘How concerned are you about your child becoming or staying overweight in the future’. The responses included ‘unconcerned’, ‘slightly concerned’, ‘concerned’, ‘fairly concerned’ and ‘very concerned.’ Both items have been used and validated in previous studies^(23,39)^.

### Statistical analysis

SPSS Statistics 24·0 and SPSS Amos 24.0 for Windows (IBM Corp) were used for data coding, cleaning and analysis. Descriptive statistics were used to describe the participants’ characteristics. The agreement between maternal weight perception and reported child weight was assessed using the Kappa statistic. Pearson’s correlation analysis was used to explore the relationships between continuous variables. All the studied variables were approximately normally distributed with skewness and kurtosis below 2·0. Parameters for the hypothetical path model were estimated using structural equation modelling via maximum likelihood estimation. The path analysis was conducted, and goodness of fit of the models was evaluated using the following indices and cut-offs: χ^2^ between 1·0 and 2·0, goodness-of-fit index (GFI) and comparative fit index (CFI) > 0·90, root mean square error of approximation (RMSEA) < 0·06, root mean square residual (RMR) < 0·08, and a small Akaike information criterion (AIC)^(49,50)^. In the final model, we controlled for demographics that were significantly associated with maternal feeding practices (*P* < 0·05). The bootstrap method was used to examine the mediating effect of weight perception and weight concern. This method has been suggested the most effective way of assessing indirect effect as it could reduce type I errors^(51)^. This method has been used in previous studies^(52,53)^. Statistical significance was set at *P* < 0·05 (two-sided).

## Results

### Demographic and socio-economic characteristics

The demographic characteristics of the participants are shown in Table 1. Two hundred nineteen (19·8 %) preschool children were reported to be overweight or obese. A few demographic variables were statistically significantly related to mothers’ feeding practices (Table 4). Maternal age was negatively associated with the use of food as a reward (*r* = –0·099, *P* < 0·001). Maternal educational level was positively associated with maternal monitoring of the child’s diet (*r* = 0·064, *P* = 0·033) and restriction of food (*r* = 0·130, *P* < 0·001). Child age was negatively associated with maternal monitoring of the child’s diet (*r* = –0·111, *P* < 0·001), applying pressure to eat (*r* = –0·067, *P* = 0·025) and restriction of food (*r* = –0·060, *P* = 0·045). Annual household income showed a positive relationship with maternal monitoring of the child’s diet (*r* = 0·084, *P* = 0·005). These variables were added to the model as covariates.

**Table 1:** Demographic characteristics of the participants (*n* 1106)

	Mean	sd
Child age	4·56	1·35
Mother’s age	34·66	3·96
Child sex	*n*	%
Boys	588	53·3
Girls	516	46·7
Child weight status		
Underweight: BMI Z-score < –2	44	4·0
Normal weight: –2 ≤ BMI Z-score ≤ 1	843	76·2
Overweight or obesity: BMI Z-score > 1	219	19·8
Mother’s weight status		
Underweight: BMI < 18·5 kg/m^2^	111	10·2
Normal weight: 18·5 kg/m^2^ ≤ BMI < 24·0 kg/m^2^	784	71·8
Overweight or obesity: BMI ≥ 24·0 kg/m^2^	197	18·0
Mother’s education level		
College or higher	872	78·8
Senior high school or below	234	21·2
Household income/year		
Above average (> CNY 300 000)	638	57·7
Below average (≤ CNY 300 000)	468	42·3

CNY: Chinese Yuan.

### Agreement between maternal perception of child weight (weight perception) and actual child weight status

Since few mothers perceived their children’s weight as very overweight or very underweight, these two statuses were combined with overweight and underweight, respectively. Three categories (perceived underweight, normal weight and overweight) were used in the final analysis. 14·1 % of mothers felt that their children weighed too much, 59·0 % reported that their children weighted a normal weight and 26·9 % felt that their children were underweight. The agreement between maternal weight perception and actual child weight status was weak (Kappa = 0·212, *P* < 0·001) (Table 2). A total of 660 (59·67 %) mothers properly perceived their children’s weight status, and 378 (34·18 %) mothers underestimated their children’s weight status. Most mothers of overweight/obese children felt their children were normal weight (*n* 106, 48·4 %) or even underweight (*n* 9, 4·1 %) (Table 2).

**Table 2:** Agreement of actual child weight status with maternal weight perception

Actual child weight status (BMI Z-score)	Maternal weight perception (*n* 1106)							
	Underweight		Normal weight		Overweight/obesity		Total	
	*n*	%	*n*	%	*n*	%	*n*	%
Underweight	26	59·1	16	36·4	2	4·5	44	3·98
Normal weight	263	31·2	530	62·9	50	5·9	843	76·22
Overweight/obesity	9	4·1	106	48·4	104	47·5	219	19·80
Total	298	26·9	652	59·0	156	14·1	1106	

Weight perception: maternal perception of child weight; Kappa = 0·212, *P* < 0·001.

### Description of variables included in the path analysis

Of the mothers, 74·1 % were not concerned about their child becoming overweight, 18·4 % reported some concern and 7·5 % reported great concern. The descriptions of weight perception, concern and feeding practices are shown in Table 3. The mean scores of weight perception (2·84) and concern (1·85) were both lower than the median level of 3. Pressure to eat and food as a reward were approximately at the median level. Slightly higher mean scores were reported for restriction and monitoring.

**Table 3:** Description of weight perception, concern and feeding practices (*n* 1106)

Variables	Range	Item numbers	Mean	sd
Weight perception	1–5	1	2·84	0·70
Weight concern	1–5	1	1·85	1·03
Pressure to eat	1–5	4	2·97	0·79
Food as a reward	1–5	2	3·38	0·76
Restriction	1–5	6	3·88	0·61
Monitoring	1–5	4	3·97	0·82

Weight perception: maternal perception of child weight; Weight concern: maternal concern about child overweight.

### Relationships between variables in the path analysis

The bivariate associations between the four feeding practices (monitoring, pressure to eat, use food as a reward and restriction), BMI Z-score, and weight perception and concern are shown in Table 4. Most of the associations were statistically significant.

### Path analysis

**Fig. 2:**
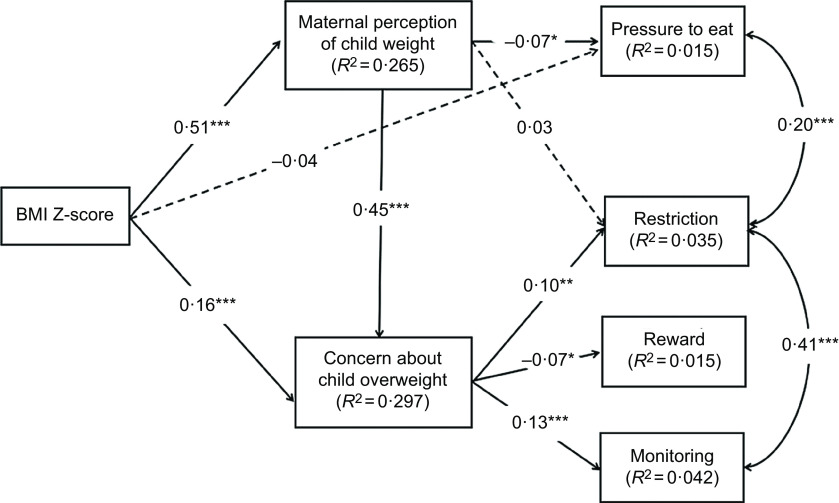
Initial model of the relationships between the study variables. Paths were adjusted for child age, maternal age, maternal education level and annual household income. Arrows represent directions. Significant paths are shown as solid lines, and non-significant paths are shown as dashed lines. ***P < 0·001; **P < 0·01; *P < 0·05

Based on the hypothesised model (Fig. 1) and the above bivariate correlation analyses, the following variables were included in the initial model: BMI Z-score, weight perception and concern, monitoring, restriction, pressure to eat, food as a reward, child age, maternal age, maternal education level, and annual family income (Fig. 2). Two pairs of error terms (restriction and monitoring, restriction and pressure to eat) were allowed to correlate as this strategy resulted in a substantial improvement in model fit. Similar approach has been used in previous studies^(54,55)^. The structural model revealed a good fit of the data: *χ* ^2^_(32)_ = 86·297 (*P* < 0·001), GFI = 0·987, CFI = 0·961, RMSEA = 0·046, RMR = 0·035 and AIC = 176·297. In this model (Fig. 2), two direct paths were non-significant. We removed the two non-significant paths, respectively, to obtain the most parsimonious model. After simplifying the model, all the remaining statistically significant paths were entered into a final model to assess the relative importance of the direct and indirect effects of BMI Z-score on maternal feeding practices.

**Fig. 3:**
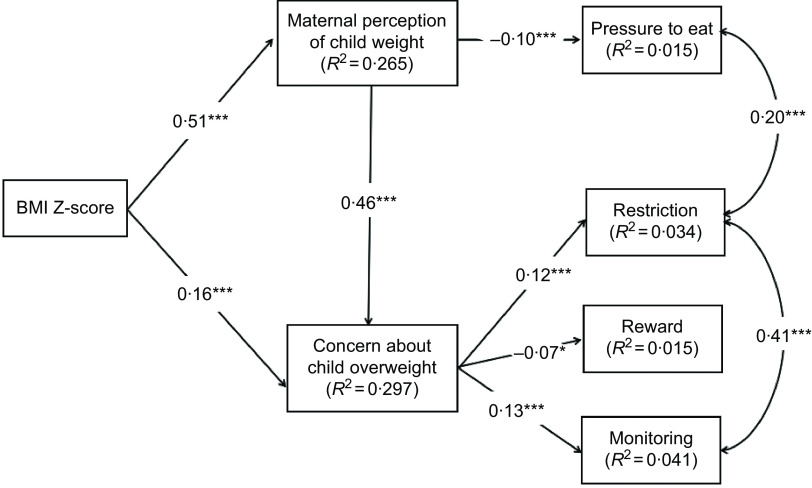
Final model of the relationships between the study variables. Paths were adjusted for child age, maternal age, maternal education level and annual household income. ***P < 0·001; **P < 0·01; *P < 0·05

The final model is illustrated in Fig. 3. It showed a better fit than the initial model, with the following parameters: *χ* ^2^_(34)_ = 89·109 (*P* < 0·001), GFI = 0·986, CFI = 0·960, RMSEA = 0·038 and RMR = 0·046. The AIC value was lower in the current model (153·109) than in the previous model. The model accounted for 1·5 %, 4·1 %, 3·4 % and 1·5 % of the variance in food as a reward, monitoring, restriction and pressure to eat, respectively. All the direct paths in the final model were statistically significant. Specifically, weight concern was associated with monitoring (standardised *β* = 0·13, *P* < 0·001), restriction (standardised *β* = 0·12, *P* < 0·001) and food as a reward (standardised *β* = –0·07, *P* < 0·05). The standardised *β* of the direct path from weight perception to pressure to eat was −0·10 (*P* < 0·001).

### Indirect effects of child weight and maternal perception of child weight (weight perception) on maternal feeding practices

The indirect effects of child weight and weight perception on maternal feeding practices are presented in Table 5. When all the variables were included in the model, both paths through a single mediation (indirect effects 1–4 in Table 5) and a serial mediation (indirect effects 5–7 in Table 5) were statistically significant. The total indirect impact on maternal feeding practices was also statistically significant (*P* < 0·05). The mediation of weight concern between weight perception and food as a reward, restriction and monitoring was also statistically significant.

**Table 4:** Correlations of the key variables (*n* 1106)

	Monitoring	Pressure to eat	Food as a reward	Restriction	BMI Z-score	Weight perception
Pressure to eat	0·142**					
Food as a reward	−0·097**	0·144**				
Restriction	0·445**	0·254**	0·026			
BMI Z-score	0·042‡	−0·084**	−0·025	0·052†		
Weight perception	0·032	−0·098**	−0·038	0·065*	0·514**	
Weight concern	0·124**	−0·058	−0·065*	0·108**	0·390**	0·528**
Maternal age	0·044	0·032	−0·099***	0·053		
Maternal educational level	0·064*	0·034	0·001	0·130***		
Child age	−0·111***	−0·067*	−0·045	−0·060*		
Annual household income	0·084**	−0·020	−0·041	0·031		

Weight perception: maternal perception of child weight; Weight concern: maternal concern about child overweight.

*P < 0·05, **P < 0·01, ***P < 0·001, †*P* = 0·086, ‡*P* = 0·160.

**Table 5:** Indirect effects of child weight and weight perception on maternal feeding practices

Indirect Effect	*β*	Boot se	Bootstrapping 95 % CI	*Z*
			Lower, upper	
1 : BMI Z-score→Weight perception→Pressure to eat	−0·034	0·010	−0·055, −0·014	3·4**
2 : Weight perception→Weight concern→Reward	−0·033	0·016	−0·066, −0·002	2·06*
3 : Weight perception→Weight concern→Restriction	0·044	0·011	0·022, 0·067	4***
4 : Weight perception→Weight concern→Monitoring	0·069	0·015	0·040, 0·100	4·6***
5 : BMI Z-score→Weight perception→Weight concern→Reward	−0·017	0·008	−0·034, −0·001	2·125*
6 : BMI Z-score→Weight perception→Weight concern→Restriction	0·022	0·006	0·011, 0·034	3·67***
7 : BMI Z-score→Weight perception→Weight concern→Monitoring	0·035	0·008	0·020, 0·052	4·375***

*n* 1106. Number of bootstrap samples for bias-corrected bootstrap CI: 10 000. Weight perception: maternal perception of child weight; Weight concern: maternal concern about child overweight.

*P < 0·05, **P < 0·01, ***P < 0·001.

## Discussion

In this study, we examined the mediating roles of maternal perception of child weight and concern about overweight on the association between child weight and four common feeding practices. The results from the structural equation modelling largely supported our hypotheses.

In this study, although the child BMI Z-score was significantly related to pressure to eat in the bivariate analysis, it was not a direct predictor in the structural equation modelling. Based on the bootstrapping results, child BMI Z-score influenced mothers’ feeding practices via three paths: two full single mediating effects of weight perception and concern and a serial multiple mediating effects of weight perception and concern. Specifically, the relationship between child BMI Z-score and pressure to eat was fully mediated by weight perception. This finding suggested that mothers of children with higher BMI Z-scores were more likely than those of children with lower BMI Z-scores to perceive their children as having a high weight, which in turn decreased the likelihood of them forcing their children to eat. Additionally, weight concern fully mediated the associations between child BMI Z-score and maternal restriction, monitoring, and use of food as a reward. In this case, mothers whose children were overweight or obese were more likely than those whose children were normal or underweight to report concerns and monitor or restrict their children’s intake rather than reward them for eating. In addition, mothers of children with higher BMI Z-scores were more likely to perceive their children as having a high weight status and express concern about their children being overweight or obese, which ultimately affected their feeding practices, such as restricting the consumption of unhealthy food.

Previous studies have assessed the correlations between caregivers’ feeding practices and weight-related variables^(14,22,25,28)^; however, few have explored their interrelationships. A population-based cohort study showed that mothers’ concerns about their children’s weight mediated the relationship between child BMI Z-score at 4 years and restrictive feeding at 10 years^(56)^. Our findings are consistent with previous evidence^(22,23,39)^ and in accordance with Costanzo and Woody’s suggestion^(57)^ that parents should use controlled feeding practices when they are concerned about their child’s weight. Therefore, although the results of this study supported our hypotheses, the mediating effect of weight perception and concern warrants further investigation.

In the current study, maternal perception of child weight was a mediator of child BMI and maternal feeding practices. This finding emphasises the importance of accurate perception of child weight. Only when mothers recognise their preschool children’s weight problems do they implement effective feeding practices to control their children’s weight. In this study, we found a high percentage of misperception of child weight, with mothers tending to underestimate their children’s weight. The results are consistent with recent findings^(58,59)^. The inability to accurately identify child overweight/obesity is problematic given that substantial evidence suggests that weight perception is critical in motivating health-related behavioural changes^(60–62)^. Thus, mothers who consider their overweight/obese children as normal weight or even underweight might not be concerned about their children being overweight or obese and therefore adopt inappropriate feeding practices, such as forcing children to eat more. This practice may further influence a child’s development and growth, especially their weight.

Although our model showed a good fit of data, the variance in feeding practices accounted for by the predictors was low and the correlations between them were weak in this study. However, our findings are consistent with previous ones^(22,39)^. For example, Gregory *et al.* ^(22)^ reported that maternal concern about child overweight only explained 2 % of the variance in restriction. This weak correlation could be due to the fact that the percentage of overweight or obese children in our study (19·8 %) were less than that in Western countries^(63,64)^. In addition, chubby children are considered healthy in traditional Chinese culture or low social economic settings^(34,36,65,66)^. Parents are usually more concerned about underweight than overweight. It is likely that mother in this study did not consider overweight a problem and thus showed a low level of concern about child overweight when compared to mothers in other countries^(18,26,39)^. It is also possible that maternal concern is more strongly related to other feeding strategies that Chinese mothers use but cannot be captured by C-CFQ (e.g. encouraging healthy eating)^(67,68)^.

To the best of our knowledge, this study was among the first to examine the structural relationships between child weight, mothers’ weight perception and weight concern, and caregivers’ feeding practices. The significant paths and well-fitted model provided further empirical support for the hypothetical model. However, this study has limited generalisability because of the sampling method used (e.g. a convenience sampling of only mothers). Related, mothers included in this study were typically college educated, and thus cannot present those from rural areas who are less educated. Although the prevalence of overweight or obese children in our study was similar to that in a large-scale study conducted in Shanghai, China^(63)^, random samples from multiple sites are needed in the future. Another limitation was the correlational design, which precluded us from making causal inferences. Additionally, self-reported information may be subject to recall bias. Finally, we focused on four common feeding practices, whereas other positive approach of feeding (e.g. rules, limits and modelling) were not assessed. Thus, longitudinal studies using comprehensive questionnaires are needed to confirm findings from this study.

## Conclusion

The model analysed in this research help to explain the interrelationships between the variables related to child weight. The results of the present study suggest that maternal perception of child weight and concern about overweight may be crucial mechanisms though which actual child weight affects mothers’ feeding practices. With respect to mothers’ feeding practices, maternal perception of child weight and concern about overweight are more relevant than actual child weight. Thus, feeding practices associated with childhood obesity should first address maternal perception of child weight. Managing concern about child overweight may be a potential intervention. Future studies should focus on correcting caregivers’ misperception about child weight. Interventions designed to improve caregivers’ accurate assessments of child weight are needed so that they can adopt appropriate feeding practices, thereby optimising child health.
